# High-Sensitivity Microwave Sensor for Liquid Characterization Using a Complementary Circular Spiral Resonator

**DOI:** 10.3390/s19040787

**Published:** 2019-02-15

**Authors:** Xingyun Zhang, Cunjun Ruan, Tanveer ul Haq, Kanglong Chen

**Affiliations:** 1 School of Electronic and Information Engineering, Beihang University, Beijing 100191, China; luckyzhang@buaa.edu.cn (X.Z.); tanveerulhaq@buaa.edu.cn (T.u.H.); yanquan1992@buaa.edu.cn (K.C.); 2 Beijing Key Laboratory for Microwave Sensing and Security Applications, Beihang University, Beijing 100191, China

**Keywords:** CCSR, dielectric constant, high sensitivity, liquid determination, low-cost, microwave sensor

## Abstract

This paper describes a low-cost, small size, and high-sensitivity microwave sensor using a Complementary Circular Spiral Resonator (CCSR), which operates at around 2.4 GHz, for identifying liquid samples and determining their dielectric constants. The proposed sensor was fabricated and tested to effectively identify different liquids commonly used in daily life and determine the concentrations of various ethanol–water mixtures at by measuring the resonant frequency of the CCSR. Using acrylic paint, a square channel was drawn at the most sensitive position of the microwave sensor to ensure accuracy of the experiment. To estimate the dielectric constants of the liquids under test, an approximate model was established using a High-Frequency Simulator Structure (HFSS). The results obtained agree very well with the existing data. Two parabolic equations were calculated and fitted to identify unknown liquids and determine the concentrations of ethanol–water mixtures. Thus, our microwave sensor provides a method with high sensitivity and low consumption of material for liquid monitoring and determination, which proves the feasibility and broad prospect of this low-cost system in industrial application.

## 1. Introduction

In recent years, food safety and water pollution have greatly attracted much attention from both research groups and industry communities due to their close relations to people’s lives and ecological environments [1,2,3,4,5]. Therefore, it is very important to identify the properties and ingredients of food products [6,7,8,9,10]. Nowadays, many sensing systems have been developed to determine the quality and characteristics of food products commonly used in daily life in a nondestructive way [11,12,13,14], which makes them useful for sample monitoring with respect to both food safety and analyses of water systems [15,16,17,18,19].

Many methods have been utilized to determine the properties of the samples to be tested [20,21,22,23,24,25,26]. A low-cost microwave vector system proposed in reference [27] has the advantage over a single transmission line to improve the sensitivity, which allows for the measurement of magnitude and phase of the material under test. In reference [28], the fat and salt contents of beef products are simultaneously measured employing microwave techniques, which have advantages such as low cost, portability, and real-time operation. Using coaxial and monopole sensors, a five-port reflectometer presented in reference [29] is applied as an available device to determine the moisture content in oil palm fruits without requiring the use of a vector network analyzer (VNA). By using a microwave resonator method, a non-contact microwave ring resonator sensor with high sensitivity is proposed in reference [30] to detect different liquid–liquid interfaces, such as water–olive oil, water–olive oil–ethanol, and rag layer samples, which makes the method attractive for the oil sands industry. A metamaterial-inspired microwave microfluidic sensor is proposed in reference [31], exploiting the advantage of a microstrip-coupled complementary split-ring resonator, which can also be used to estimate the dielectric properties of the liquid samples. A novel wireless, high-resolution, resonant-based microwave sensor is presented to enable high-resolution sensing in non-accessible applications, while minimizing the battery usage [32]. However, some of the sensors mentioned above suffer from their large size and required integration with other devices [27,32], such as power detectors, DC sources, and data acquisition boards or the use of larger quantities of samples for testing, because of their large sensing areas. Besides, the identification of different substances has only been reported on a low scale so far.

In this paper, a high-sensitivity microwave system for liquid monitoring using a Complementary Circular Spiral Resonator (CCSR) is proposed. The system is designed to operate at around 2.4 GHz and is easy to integrate with other platforms. Through careful experimentation and analysis, the proposed sensor has the ability to identify some common liquids and determine the concentrations of ethanol–water mixtures. Meanwhile, the dielectric constant of materials under test can also be obtained by building a relatively accurate model in a High-Frequency Simulator Structure (HFSS). By using the dielectric constant, fitting equations are calculated to determine a solution type and its corresponding concentrations, according to the measured resonant frequency of the materials present. The proposed system has advantages, such as a small size, low fabrication and measurement costs, a simple structure, easy operation, measurement accuracy, a very low sample consumption, and high sensitivity, which makes the system attractive for sensing applications [33,34].

## 2. Materials and Methods

### 2.1. Sensor Design

**Figure 1:**
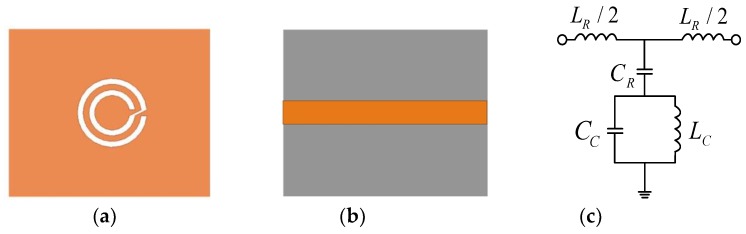
(**a**) Top view and (**b**) bottom view of the microwave sensor based on the Complementary Circular Spiral Resonator (CCSR); (**c**) equivalent circuit of the sensor.

**Figure 2:**
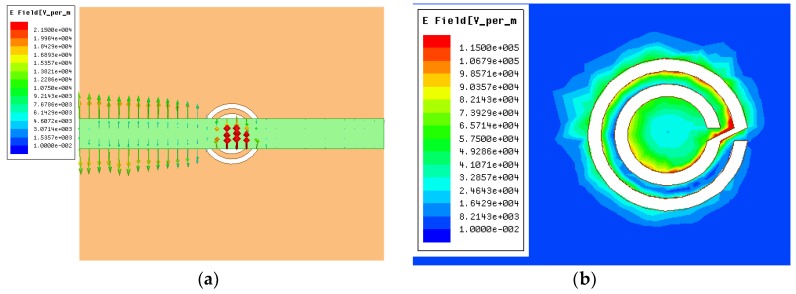
(**a**) Excitation of the CCSR with the help of an electric field generated by the microstrip transmission line; (**b**) distribution of the electric field in the CCSR at resonance.

**Figure 3:**
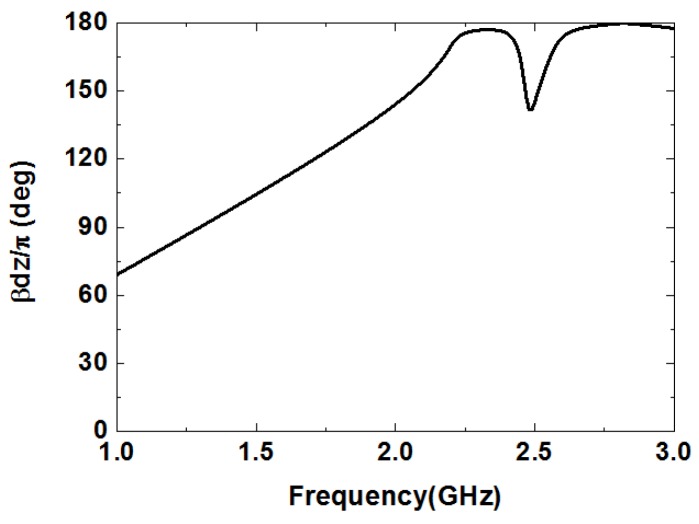
Dispersion diagram of the microwave sensor based on the CCSR.

The microwave sensor consists of a microstrip transmission line and a CCSR which is a complementary counterpart to a circular spiral resonator (CSR). The equivalent circuit model of the proposed sensor is shown in Figure 1 [35]. In the equivalent circuit, $L_{R}$ represents the inductance of the microstrip line, $C_{R}$ is the coupling capacitance between the microstrip line and the ground, $C_{C}$ and $L_{C}$ are the capacitance and inductance of the CCSR, which is described by means of a parallel tank. The CCSR is excited by an electric field to generate resonance. Here, the electric field is provided by the microstrip transmission line right above the resonator, as shown in Figure 2a. When the electric field collides with the CCSR, it generates resonance at the operation frequency. The distribution of the electric field is shown in Figure 2b. Figure 3 shows the dispersion characteristics of the proposed microwave sensor. There is a nonlinear relation between the propagation constant β and the frequency, which implies that the values of permittivity and permeability depend on the operating frequency of the CCSR [36]. The ripples in the dispersion diagram are due to negative permittivity of the CCSR.

### 2.2. Measurement Setup and Device Performance

**Figure 4:**
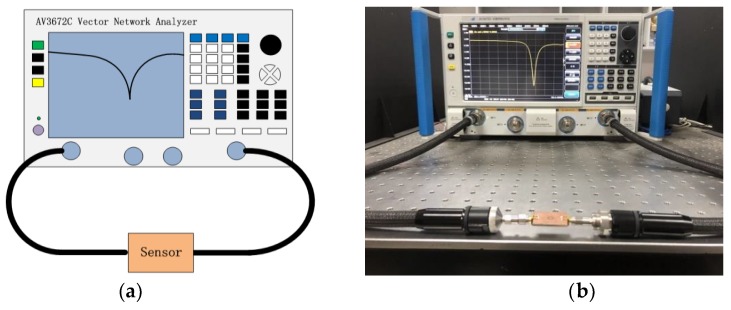
(**a**) Schematic diagram of the proposed microwave sensing system used to identify different materials and determine the corresponding concentration; (**b**) photograph of the setup of the measuring experiment.

**Figure 5:**
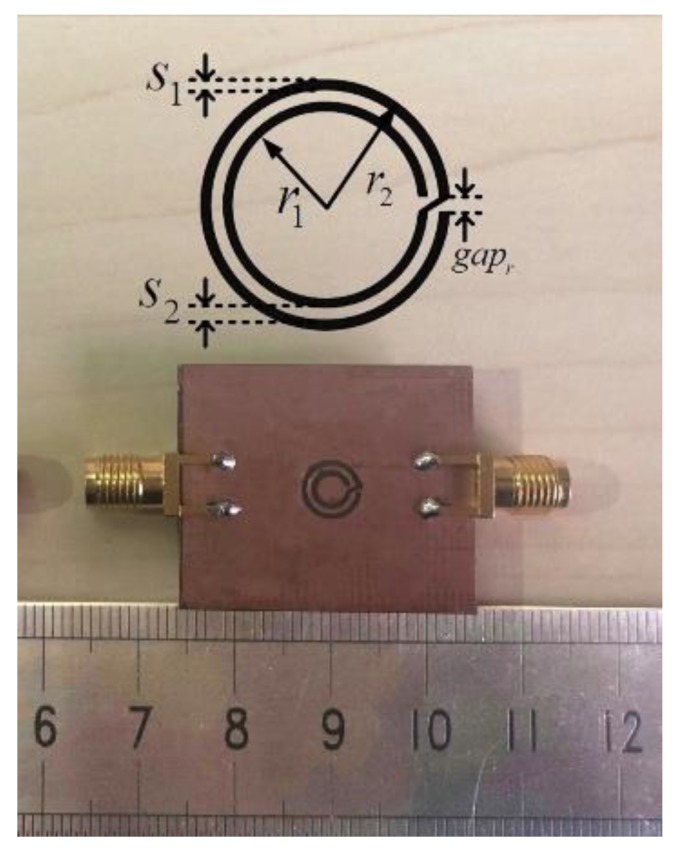
Photograph of the CCSR for liquid monitoring with its key dimensions.

In our experiment, we fabricated the microwave biosensor based on the CCSR, employing the hot transfer technology. The schematic diagram of the developed microwave biosensor for liquid determination and its deployment are shown in Figure 4. A vector network analyzer (AV3672C, 10 MHz–43.5 GHz), shown in Figure 4, was used to measure the transmission response of the proposed sensor. All experiments were carried out at a temperature of 26 °C and a humidity of 10.5%. A photograph of the proposed microwave sensor, which was fabricated on FR4 substrate with a relative permittivity of 4.4 and was 25 mm × 30 mm in size, is shown in Figure 5. The substrate thickness was 1.6 mm. The copper metallization for the ground plane and 50 Ω microstrip line was 35 μm. For the CCSR, the geometrical parameters are optimized as follows: r_1_ = 2 mm, r_2_ = 3 mm, S_1_ = S_2_ = gap_r_ = 0.5 mm.

#### 2.2.1. Device Characterization

**Figure 6:**
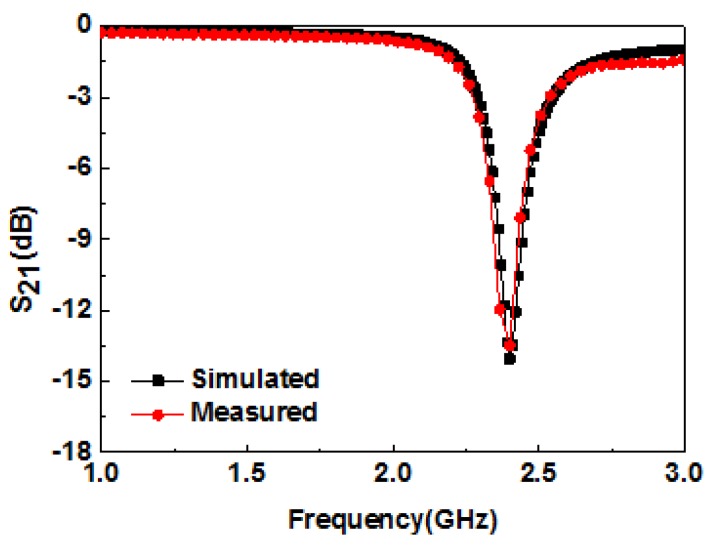
Simulated and measured resonance characteristics of the CCSR.

The measured and simulated results of the transmission coefficient of the microwave biosensor are shown in Figure 6. It is observed that the results of simulation and measurement are in agreement with each other. The difference of amplitude between simulated and measured results is mainly due to fabrication and measurement errors. Detailed data of the simulated and measured results are given in Table 1.

#### 2.2.2. Sensitivity of Different Positions 

**Figure 7:**
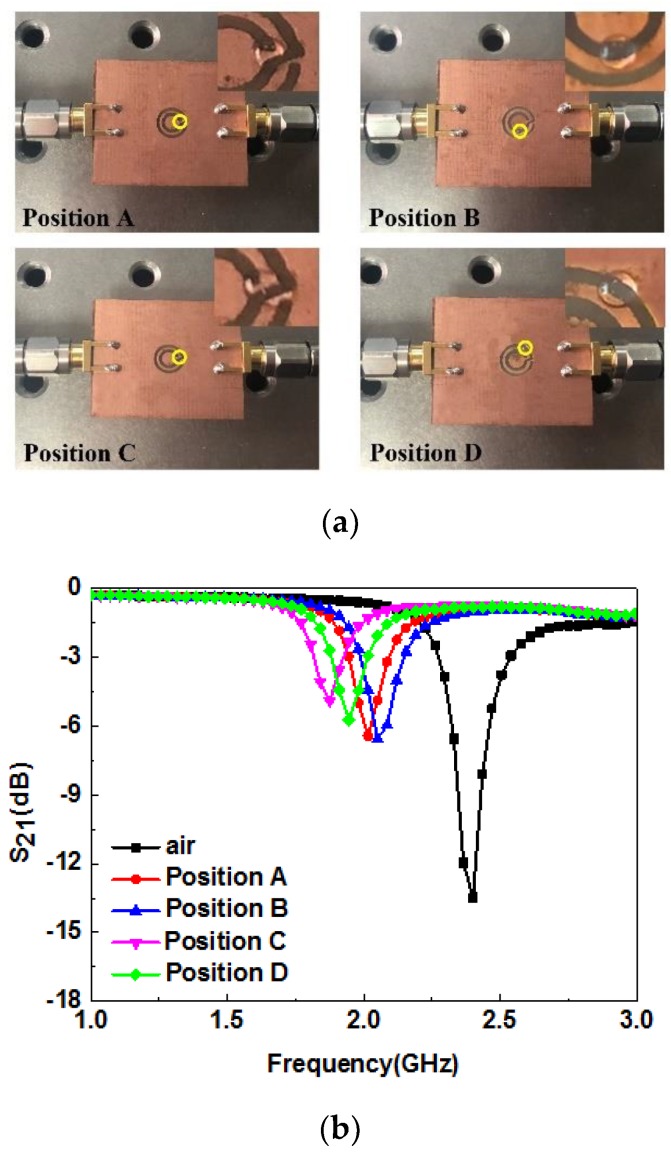
(**a**) Photograph of the sensors with the liquid sample in different positions; (**b**) measured resonance characteristics of deionized water in different positions.

The sensitivity of different positions of the proposed microwave sensor was investigated. Equal amounts of deionized water (0.3 μL) were put in positions A, B, C, and D of the proposed sensor, using a precision pipette (Eppendorf, Research plus, 0.1−2.5 μL), as shown in Figure 7a. Figure 7b shows the measured transmission response of the test sample in different sensor positions. When the liquid sample was put in position C, the frequency deviation relative to the air was the most obvious, which is consistent with the presence of a strong electric field at position C, as shown in Figure 2b. The transmission characteristics of the microwave sensor with deionized water in different positions are given in Table 2. Thus, position C was chosen as the most sensitive position for the following experiments.

## 3. Measured Results with Different Samples

**Figure 8:**
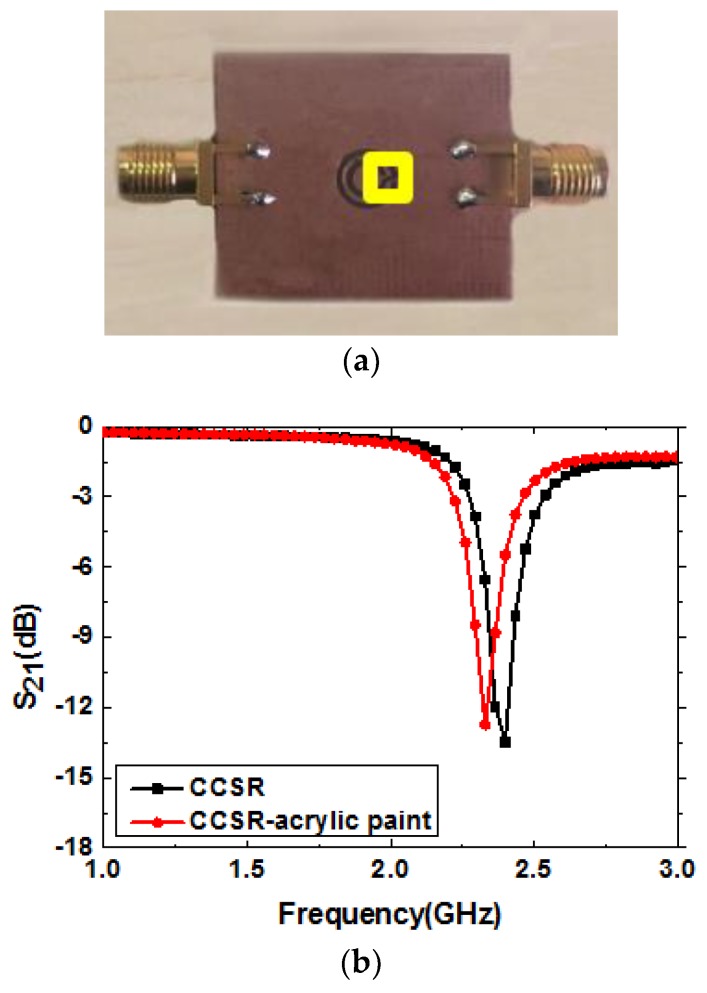
(**a**) Photograph of the post-processing sensor with the channel drawn by acrylic paint; (**b**) measured resonance characteristics of the sensor with the channel drawn on the substrate.

To verify the feasibility of the presented measurement system, the experiment was divided into two parts. First, an experiment was done to prove that the system is sensitive to the different liquid samples to be tested. Second, having confirmed the sensitivity of the proposed sensor to different liquids, the ability of the developed system to determine different concentrations of the same substance, namely a mixture of ethanol and water, was also been investigated. Notably, in order to ensure a constant shape of the liquid sample across the sensing section, a channel was drawn around position C using acrylic paint, as shown in Figure 8a. It should also be mentioned that the pipette was set to 2 μL for all measurements to guarantee the accuracy and consistency of the experiments. When 2 μL of a liquid sample was dropped into the channel, the liquid was uniformly tiled in the square area within the yellow line. Therefore, the hydrophobicity of liquids did not affect the test results because of the constant amount and shape of the sample. The measured results of the sensor with the channel drawn by acrylic paint are shown in Figure 8b. It was observed that the resonance frequency was slightly affected when acrylic paint was drawn in the sensor, but this did not compromise the results since the channel position remained unchanged in all of the following measurements.

### 3.1. Different Liquids

**Figure 9:**
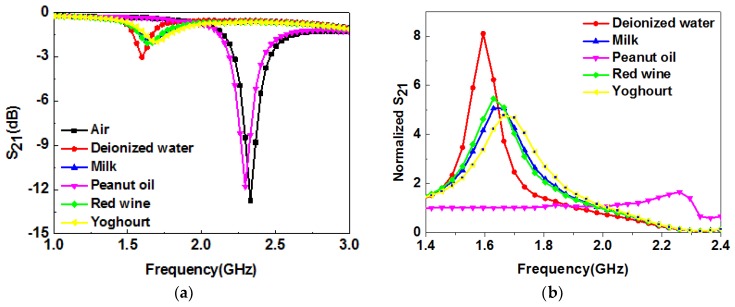
(**a**) Measured resonance characteristics of different liquids; (**b**) measured resonance characteristics in (a) divided by the reference signal.

The developed system can be used for the determination of liquids’ properties. Some familiar liquids, such as deionized water, milk, peanut oil, red wine, and yogurt were selected for analysis. In order to minimize the impact of contamination and humidity from the previous test samples, the channel area was wiped with alcohol using a clean cotton ball, the alcohol was let evaporate for 30 s, and then the next liquid sample was dropped. Each set of measurements was carried out at least 15 times to reduce measurement errors and ensure accuracy. The measured transmission coefficients, $S_{21}$, of all samples are presented in Figure 9a. The curve of air is a reference signal, and the other curves containing solution information are the sensing signals. The data shown in Figure 9a were processed as follows: the amplitudes of the sensing signals were divided by that of the reference signal. The variations of all sensing signals, relative to the reference signal, are shown in Figure 9b. It can be observed that there is a big difference in the varied transmission response of the liquids under test, which shows that the developed sensor can be used to distinguish different liquids.

### 3.2. Ethanol–Water Solution Measurements

**Figure 10:**
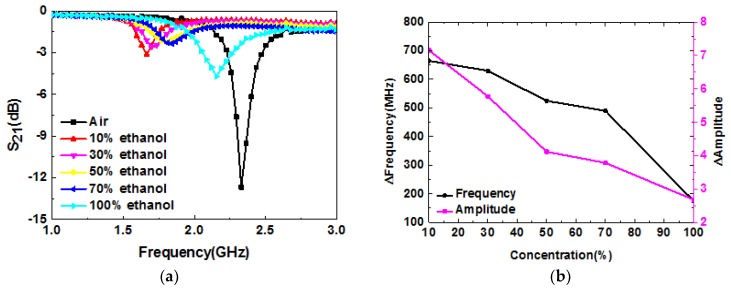
(**a**) Measured resonance characteristics of ethanol–water mixtures at different concentrations; (**b**) relative variations of frequency and amplitude, compared with the reference.

Having experimentally verified the sensitivity of the developed system to different materials, the capability of determining different concentrations of a solution was tested. The sample used fort this experiment was an ethanol–water mixture. The concentration of the ethanol–water mixture is defined by the volume ratio of anhydrous ethanol to that of the deionized water. Figure 10a shows the measured results of ethanol–water mixtures at different concentrations. As seen, the frequency shift was about 490 MHz as the ethanol volume ratio increased from 0% to 100%, which indicates that the sensitivity of our sensor is 1.225 times that of the system reported in reference [31]. Furthermore, compared with planar microwave sensors [37] and 3-D printed microfluidic channels [38] for sensing ethanol–water concentrations, our sensors have the highest sensitivity. The detailed comparisons are presented in Table 3. The relative variations of frequency and amplitude, compared with the references, are shown in Figure 10b. As it can be observed, the Δamplitude decreased with the concentration increasing from 10% to 100%. Moreover, it is evident that the Δfrequency monotonically varied with the concentration of the ethanol–water mixture; therefore this parameter can be used to determine the concentration of different ethanol–water mixtures. 

## 4. Simulation and Analysis of the Dielectric Constant

**Figure 11:**
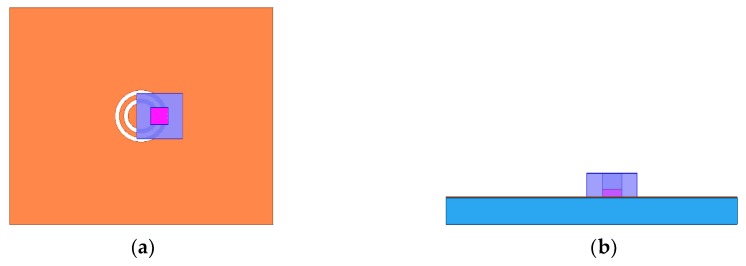
Simulation model of the proposed sensor using a High-Frequency Simulator Structure (HFSS) with the material under test filling the channel drawn by acrylic paint (**a**) Top view; (**b**) side view.

In the previous section, it is evident that different liquids and ethanol–water mixtures with varied concentrations can easily be distinguished using the developed microwave sensor. The resonance frequency and transmission characteristics can also be used for calculating the dielectric constant of materials. Therefore, some unknown liquid samples with unique permittivity as well as their concentrations can be easily determined by the proposed system. According to the position and shape of the liquids in the experiments, a relatively accurate model that takes into account the channel using a HFSS, is shown in Figure 11. Violet and magenta represent the channel and material under testing, respectively. The height, width, and length of the channel were 1.5 mm, 2 mm, and 2 mm. The height of the material was 0.5 mm. By changing the dielectric constant of a liquid, making it fit the measurement results as much as possible, the obtained value is very close to the real dielectric constant of the liquid.

### 4.1. Different Liquids

**Figure 12:**
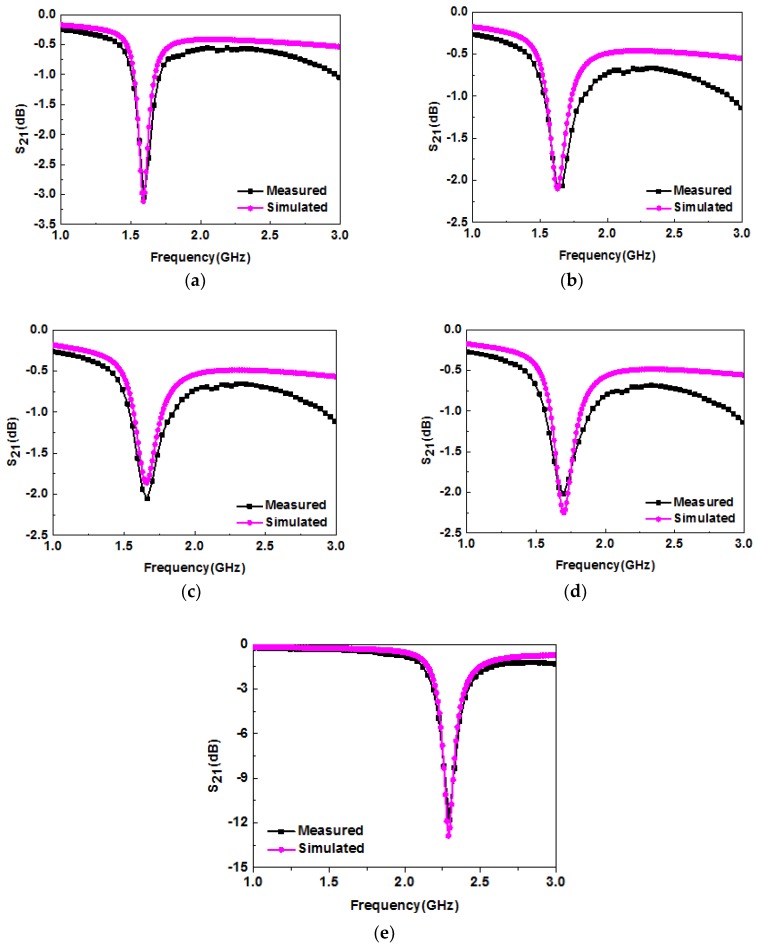
Simulated and measured resonance characteristics of (**a**) deionized water; (**b**) red wine; (**c**) milk; (**d**) yogurt; (**e**) peanut oil.

Using the method demonstrated above, the simulated and measured results for five liquid samples are shown in Figure 12. The obtained dielectric constants of deionized water, red wine, milk, yogurt, and peanut oil were 72, 66, 62, 57, and 2, respectively. The derived dielectric constants of deionized water, red wine, and milk agreed well with those reported in the literature [27,39,40], which indicates the validity and accuracy of the experiment to a certain extent. In order to identify unknown liquids, a parabolic equation was used to fit the relationship between resonant frequency and dielectric constant. The formula with three fitting coefficients is as follows [41]:

(1) $$f_{r,MUT}(\varepsilon_{r})=A_{1}+A_{2}\varepsilon_{r}+A_{3}\varepsilon^{2}{}_{r}$$

By using curve fitting, the coefficients of (1) can be derived. The fitting equation becomes

(2) $$f_{r,MUT}(\varepsilon_{r})=481.654-366.462\varepsilon_{r}+68.613\varepsilon^{2}{}_{r}$$

**Figure 13:**
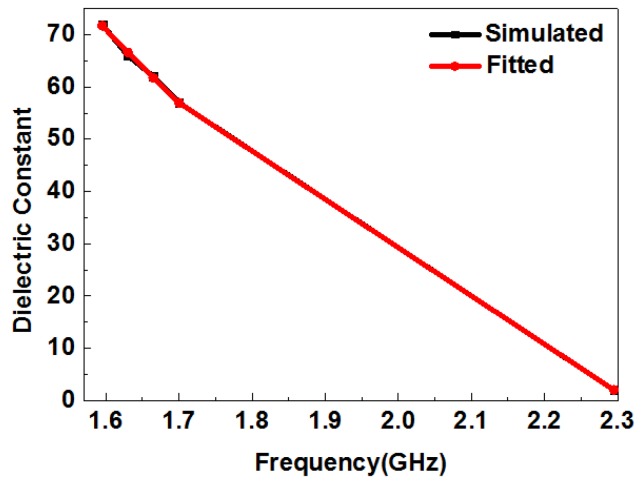
Simulated and fitted curve of the resonant frequencies of different liquids and their dielectric constants.

The simulated and fitted dielectric constants of liquid samples are shown in Table 4. We measured each liquid sample at least 15 times for accuracy. The results were basically the same for all samples. Therefore, here, we only show the results for red wine as an example of the deviations measured. The standard deviation of the measured resonant frequency was approximately equal to 1.630 ± 0.005 GHz. According to the fitting equation and simulation, the fitting permittivity became 66 ± 0.85, which indicates the reliability of the data. The extracted permittivity of milk at room temperature agreed well with the measurement obtained using a commercial dielectric probe kit [40]. Figure 13 shows the simulated and fitted curve of the resonant frequency of different liquids and their dielectric constants. From Figure 13, we can see that the fitted curve and the simulated curve are in a good agreement, which illustrates the feasibility of this formula. When different liquids are tested with our device, the resonant frequency can be measured, and then the dielectric constant of a liquid sample is calculated by this formula. Thus, unknown test materials can be successfully identified by our device.

### 4.2. Ethanol–Water Solution

**Figure 14:**
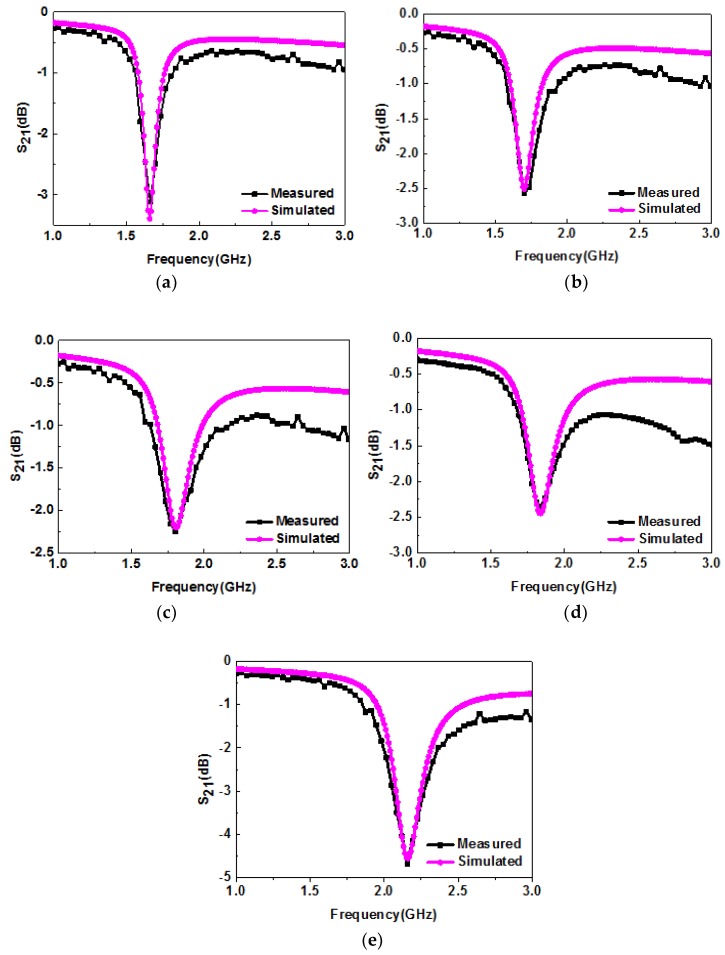
Simulated and measured resonance characteristics of ethanol–water mixtures at (**a**) 10%; (**b**) 30%; (**c**) 50%; (**d**) 70%; (**e**) 100% concentrations.

Figure 14 shows the simulated and measured transmission response of the sensor in ethanol–water mixtures at different concentrations. When the simulation results were in good agreement with the measured results, the obtained permittivity of ethanol–water solutions at concentrations of 10%, 30%, 50%, 70%, and 100% was 60, 55, 43, 40, and 11, respectively. The obtained dielectric constants of ethanol–water mixtures at different concentrations are basically consistent with those reported in the literature [31], which confirms the reliability of this experiment. Similar to what reported previously, the fitting equation is derived as follows:

(3) $$f_{r,MUT}(\varepsilon_{r})=385.007-269.680\varepsilon_{r}+44.606\varepsilon^{2}{}_{r}$$

**Figure 15:**
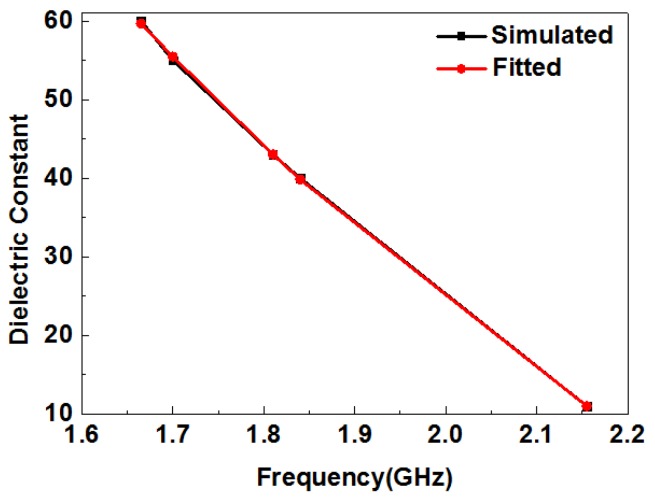
Simulated and fitted curve of the resonant frequencies of ethanol–water mixtures at different concentrations and their dielectric constants.

Table 5 shows the simulated and fitted dielectric constants of ethanol–water mixtures at different concentrations. The extracted permittivity of ethanol–water solutions at different concentrations at room temperature agreed with those obtained using a commercial dielectric probe kit [42]. Figure 15 shows the simulated and fitted curve of the resonant frequencies of ethanol–water mixtures at different concentrations and their dielectric constants. The two curves are almost the same, thus the fitting equation has a high degree of accuracy. By measuring the resonance frequency of an ethanol–water solution at an unknown concentration, the dielectric constant can be calculated, which is then used to estimate the concentration of the ethanol–water mixture.

## 5. Conclusions

A small-size, low-cost, and efficient microwave liquid sensor operating at 2.4 GHz is proposed and validated in this paper. By putting the liquids under test on the connected region of the gap between the inner and the outer ring, where a very strong and localized electric field on resonance exists, the measured transmission response changes greatly. Through careful experimentation, some common liquid samples and the concentrations of ethanol–water mixtures can be determined by the proposed device. A simulation model was built using a HFSS to obtain the dielectric constants of liquid samples, then fitting equations were used to identify different liquids and determine the concentrations of ethanol–water solutions. The proposed sensor has advantages, being inexpensive, reliable, easy to operate, and highly sensitive, and has potential applications in liquid monitoring and quality control. Future work will mainly focus on improving the Q factor of the sensor in order to distinguish different materials more precisely.

## Figures and Tables

**Table 1:** Measured and simulated results of the microwave sensor.

Results	*f* (GHz)	Notch Depth
Simulated	2.400	−14.05 dB
Measured	2.400	−13.47 dB

**Table 2:** Transmission characteristics of the microwave sensor with deionized water in different positions.

Results	*f* (GHz)	Notch Depth
Air	2.400	−13.47 dB
Position A	2.015	−6.44 dB
Position B	2.050	−6.56 dB
Position C	1.875	−4.89 dB
Position D	1.945	−5.73 dB

**Table 3:** Comparison of sensitivity between our sensor and other microwave sensors.

Sensors	Permittivity Varying Range or Ethanol Concentration in Water (%)	Resonant Frequency Shift
[31]	0%–100% concentration	400 MHz
[37]	From 11 to 60	400 MHz
[38]	3%–100% concentration (almost the same as we used)	230 MHz
ours	From 11 to 60 (0%–100% concentration)	490 MHz

**Table 4:** Measured resonant frequencies and dielectric constants of liquid samples.

Liquid Sample	*f* (GHz)	$\mathrm{Simulated}\;\varepsilon_{r}$	$\mathrm{Fitted}\;\varepsilon_{r}$
Deionized water	1.595	72	71.70
Red wine	1.630	66	66.62
Milk	1.665	62	61.71
Yoghourt	1.700	57	56.96
Peanut oil	2.295	2	2.01

**Table 5:** Measured resonant frequencies and dielectric constants of ethanol–water mixtures at different concentrations.

Concentration (%)	*f* (GHz)	$\mathrm{Simulated}\;\varepsilon_{r}$	$\mathrm{Fitted}\;\varepsilon_{r}$
10	1.665	60	59.65
30	1.700	55	55.46
50	1.810	43	43.02
70	1.840	40	39.82
100	2.155	11	11.00
